# Three-dimensional reconstruction and virtual reposition of fragments compared to two dimensional measurements of midshaft clavicle fracture shortening

**DOI:** 10.1186/s12891-022-05173-4

**Published:** 2022-03-07

**Authors:** Mehmet Öztürk, Emilie Paulin, Caecilia Charbonnier, Elise Dupuis-Lozeron, Nicolas Holzer

**Affiliations:** 1 Division of Orthopedic and Trauma Surgery, Geneva University Hospital, Geneva, Switzerland; 2 Department of Radiology, Geneva University Hospital, Geneva, Switzerland; 3 Artanim Foundation, Medical Research Department, Geneva, Switzerland; 4 Department of Clinical Epidemiology, Geneva University Hospital, Geneva, Switzerland

**Keywords:** Clavicle fracture, Shortening, Computed tomography, 3D analysis

## Abstract

**Background:**

Midshaft clavicle fracture shortening measurement is a reported key element for indication to surgical management and reporting of clinical trials. Determination of pre-fracture clavicle length for shortening measurement remains an unresolved issue. The purpose of the study was to assess accuracy of a novel technique of three-dimensional reconstruction and virtual reposition of bone fragments (3D-VR) for determination of pre-fracture clavicle length and measurement of shortening.

**Methods:**

Accuracy of 3D-VR measurements was assessed using 5 synthetic bone clavicle fracture models. Measurements were compared between caliper and 3D-VR technique measurements. Correlation between 3D-VR and 2D measurements on standard radiographs was assessed on a cohort of 20 midshaft fractures. Four different methods for 2D measurements were assessed.

**Results:**

Mean difference between caliper measurements and 3D-VR was 0.74 mm (95CI = − 2.51;3.98) (*p* = 0.56) on synthetic fracture models. Mean differences between 3D-VR and standard radiograph shortening measurement methods were 11.95 mm (95CI = 7.44;16.46) for method 1 (Jeray et al.) and 9.28 mm (95CI = 4.77;13.79) for method 2 (Smekal et al.) (*p* < 0.05). Differences were − 1.02 mm (95CI = − 5.53;3.48) for method 3 (Silva et al.) and − 2.04 mm (95CI = − 6.55;2.47) for method 4 (own method). Interobserver correlation ranged between 0.85 and 0.99. A false positive threshold of 20 mm was measured by the two observers in 25% of the case according to method of method 1, 30–35% with method 2, 15% with method 3 et al. and 5–10% with the method 4.

**Conclusion:**

3D VR is accurate in measuring midshaft clavicle fracture length and shortening. Two dimensional measurements may be used for approximation of clavicular shortening.

## Introduction

Clavicle fractures are very common in adults, accounting for 5 to 10% of all fractures [1, 2]. A large majority (70–80%) occurs at the middle third [2, 3]. Reported indications for surgical management of midshaft clavicle fracture include open fractures, skin threats, polytrauma patients, absence of contact between fragments, a verticalized intermediate fragment and a shortening of 14 to 20 mm [4–7]. Quantification of shortening is commonly achieved by measuring distances (overlap) between fragments on standard two-dimensional (2D) radiography [8, 9]. Two dimensional projections of the complex 3D structure of fractured clavicle may though lead to image distortion and 2D measurement have been shown to be inaccurate compared to 3D measurements on computed tomography (CT) [10, 11]. Alternative methods relying on comparison of fractured clavicle length with contralateral side are not recommended. Cadaveric studies have demonstrated difference up to 15 to 20 mm in length between the two clavicles of the same individual [12, 13]. Three-dimensional analysis of clavicle fracture length after 3D reconstruction and virtual reposition of bone fragments (3D-VR) represents an innovative approach overcoming current limitations encountered in clavicle fracture shortening assessment.

Our primary objective was the determination of the accuracy of 3D-VR on synthetic bone models allowing direct as well as digital measurements. Our secondary objective was the assessment of correlation between 3D-VR measurements and 2D measurements in a series of midshaft clavicle fracture patients imaged by standard radiography and CT. Four methods measuring 2D clavicle shortening were assessed.

## Methods

### Three-dimensional virtual reposition (3D-VR)

**Fig. 1:**
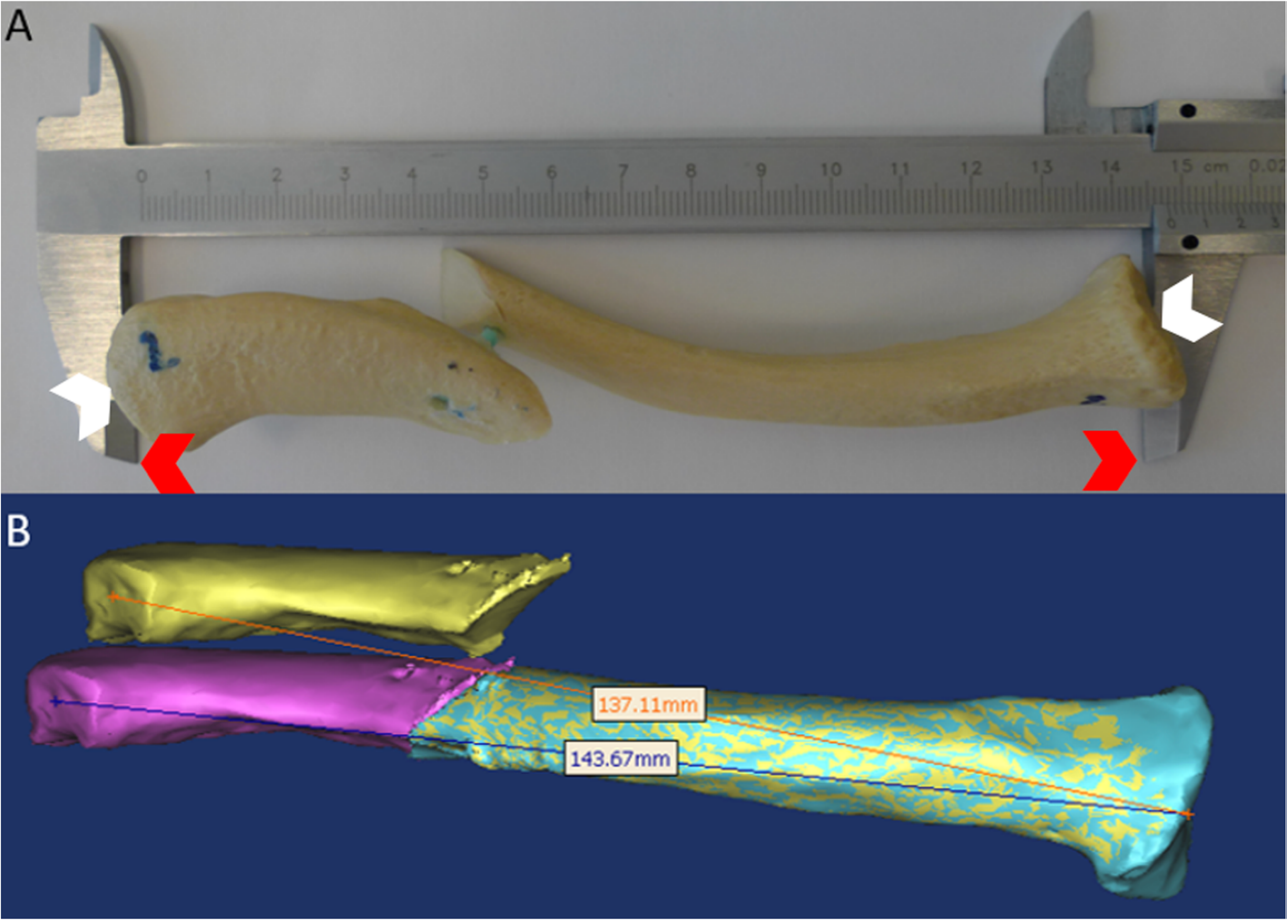
**A** Fracture models were created on standard synthetic bone models and rigidly fixed. Clavicle length was directly measured positioning the point (red arrow) of a handheld caliper at the articular center (white arrow) of intact and fractured models for calculation of shortening. **B** Three-dimensional reconstruction where obtained and measures were repeated on fractured and virtually reduced bone fragments

Mid-third clavicle fractures were created on 5 synthetic clavicles (PR0627.1, Synbone®, Zizers, Switzerland) with a surgical oscillating saw in oblique and transverse patterns. Fragments were stabilized in a displaced position by fixation of interfragmentary plastic rods. Clavicle length was manually recorded using a handheld millimetric caliper (Lux® Comfort caliper) and compared to a non-fractured synthetic bone model (Fig. 1A). Each synthetic clavicle was measured twice by three different observers to assess the accuracy of the manual measurement.

CT images of bone models were acquired (cut thickness of 0.3 mm). Images were post-processed and clavicle models were reconstructed in 3D using Mimics® software (Materialize NV, Leuven, Belgium). Each fracture was digitally reduced with the software by alignment of the reduction criteria used during surgical procedure. Pre- and post-reduction length measurement were obtained for computation of shortening measurement (Fig. 1B).

### Clinical study design

We retrospectively looked in our hospital database to retrieve polytrauma patients displaying midshaft clavicle fracture who had been imaged by both anteroposterior (AP) clavicle X-Ray and thoracoabdominal CT with a cut thickness of 1.25 mm. Exclusion criteria where pediatric patient, X-Ray that didn’t meet true AP criteria and CT images that couldn’t be reconstructed in 3D.

### Clavicle shortening measurements

**Fig. 2:**
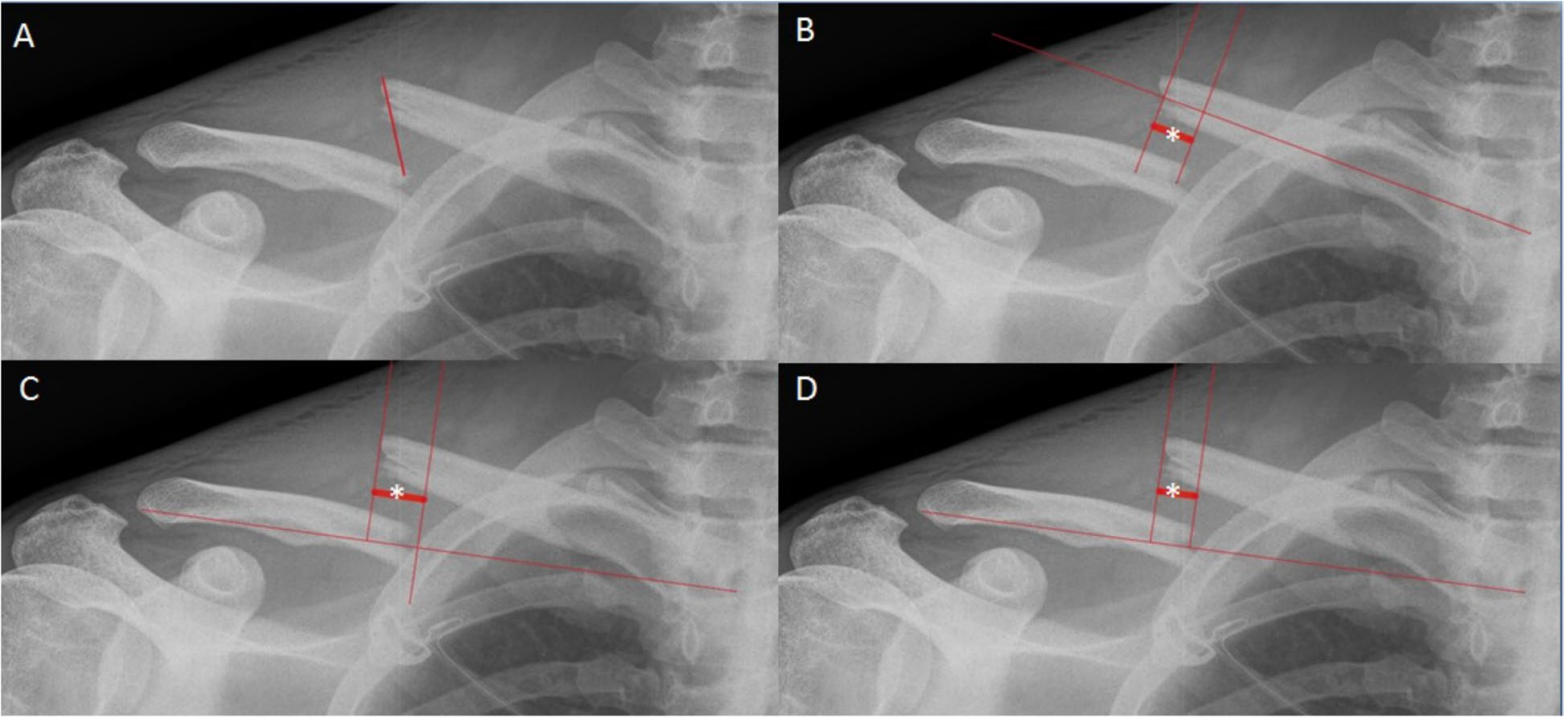
Radiographic midshaft clavicle fracture shortening measurement methods: **A** Jeray et al., **B** Silva et al., **C** Smekal et al. and **D** HUG method. See text for description

Midshaft clavicle fracture Shortening was measured on conventional radiographs using four methods described in the literature. 1) Method by Jeray et al. measures the distance between reduction criteria on each fragment (Fig. 2A) [14]; 2) Method by Silva et al. draws the longitudinal axis of each fragment and measures the distance between two perpendicular lines to the proximal fragment passing by the extremity of each axis (Fig. 2B) [15]; 3) Method by Smekal et al. measures the distance between two perpendicular lines to the whole fractured clavicle axis and passing by the most extreme point of each fragment (Fig. 2C) [16]; 4) Our own method, named HUG, which measures the distance between two perpendicular lines to the axis of the clavicle passing by two reduction criteria on each fragment (Fig. 2D). This allows not to rely only on the overlapping of fragments but on landmarks analogous to the one used during surgery. All measurements were performed on the Osirix®software (Geneva, Switzerland) by two observers, a radiologist specialized in osteoarticular imaging and an experienced orthopedic surgeon.

Shortening was assessed on same cases imaged by CT using 3D-VR technique and compared with 2D radiographic measurements.

### Statistical analysis

Shortenings measured by CT and true shortening values measured on synthetic bone models were compared using a Student t-test. Considering the shortening measured by CT on each patient as the true measure of shortening, comparison of the errors made by the four measurement methods on radiographs were done using a mixed-effect linear model with a random intercept for each patient and a fixed effect for the two raters and the four measurement methods. Bland-Altman plot analysis was carried with the gold-standard value expressed on the X axis (shortening measured by CT) [17].

Inter-rater reliability of the four measurement methods on radiographs were evaluated using Intra-Class Correlation coefficient (ICC) from a mixed-effect linear model with a random intercept for each patient and a fixed effect for the two raters [18].

All analyses were done using R, version 3.5.2 (R Core Team, 2018) and a 2-sided statistical significance threshold of 0.05.

## Results

### Clavicle length measurement of synthetic bones

Manual measurements of the 5 synthetic clavicle fracture models showed a mean standard deviation of 0.67 mm (min = 0.00 mm; max = 1.21 mm).

### Three-dimensional virtual bone fragments reposition

When measuring clavicle length on synthetic bones, a mean difference between the CT reconstruction and manual measurements of 0.736 mm (95CI = − 2.51;3.98) was recorded. T-test showed non-significant difference between manual and 3D-VR measurements (*p* = 0.56).

### Patients

A review of the trauma database of our service allowed us to retrieve data from 21 patients who suffered a fracture of the middle third of the clavicle and who got standard radiographs and a CT imaging. Two patients didn’t meet the criteria of a true anteroposterior radiograph and were excluded. Among the patients retained, one suffered bilateral fractures, and both were used individually. There were 3 women and 16 men. All patients were adults and mean age was 48.53. The images were anonymously analyzed.

### Clavicle shortening measurements

**Fig. 3:**
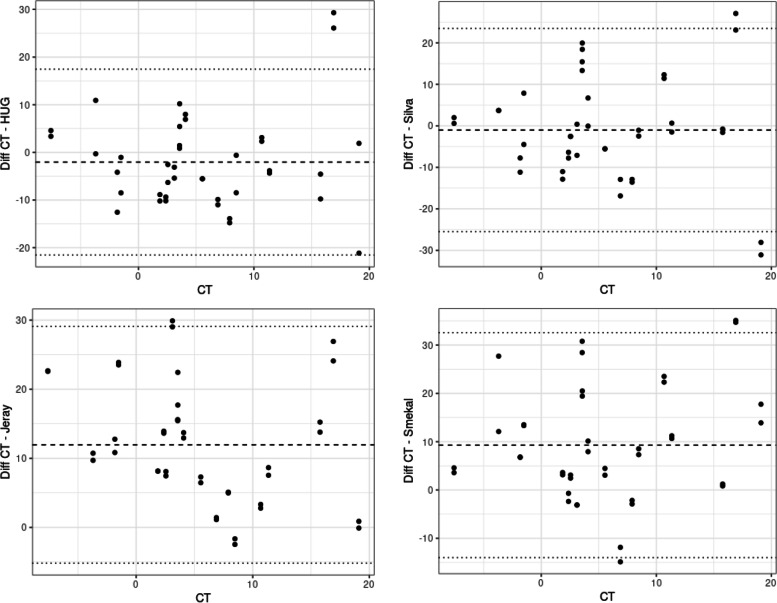
Graphic representation of the Bland-Altman analysis. On the X axis, the shortening measurement by CT. On Y axis, the differences between each radiological method and CT measurement. Values are in millimeters

The methods according to Jeray et al. and Smekal et al. differed significantly from CT measurements: 11.95 mm (95CI = 7.44;16.46, *p* < 0.0001) and 9.28 mm (95CI = 4.77;13.79, *p* = 0.0002), respectively. Methods according to Silva et al. and personal method (HUG) method did not significantly differ from CT measurements: − 1.02 mm (95CI = − 5.53; 3.48, *p* = 0.645) and − 2.03 mm (95CI = − 6.55; 2.47, *p* = 0.3626) respectively. Bland-Altman plot analysis showed differences closest to 0 for these two methods (Fig. 3). Inter-rater reliability was globally good for all methods, (ICC_Jeray_ = 0.99; ICC_Smekal_ = 0.97; ICC_Silva_ = 0.97; ICC_HUG_ = 0.85).

**Fig. 4:**
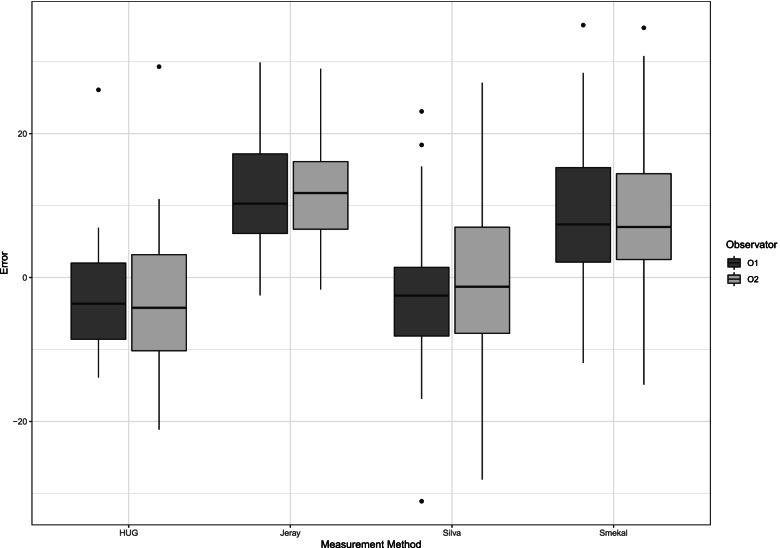
Distribution of differences between 3D-VR and 2D methods for midshaft clavicle fracture shortening measurement (Mean, Confidence interval). Thresholds of 20 mm of shortening indicated, positive value represent shortening (*n* = 20)

The measurement on CT, did not show value above the 20 mm surgical threshold. Two dimensional methods all showed occurrences where 3D-VR measurements exceeded a 20 mm threshold (Fig. 4). Number of cases per method, where 3D-VR measurements show a value under the threshold of 20 mm when 2D measurements show a value above the threshold, are reported in Table 1.

**Table 1:** Number of false-positive cases comparing 3D-VR measurements with 2D methods measurements for a 20 mm surgical threshold (*n* = 20)

	Observer 1	Observer 2
**HUG method**	2 (10%)	1 (5%)
**Jeray et al.**	5 (25%)	5 (25%)
**Silva et al.**	3 (15%)	3 (15%)
**Smekal et al.**	7 (35%)	6 (30%)

## Discussion

This study showed that 3D-VR was accurate in measuring shortening of midshaft clavicle fracture, with mean difference of 0.736 mm (95CI = − 2.51;3.98) compared to manual measurement. When compared to 2D radiographic methods, a partial correlation was observed. The method proposed by Silva et al. and the HUG method were the only measurements correlating significantly with the CT measurements – the HUG method being the less sensitive to false-positive surgical indication. There was no evidence of impact of fracture pattern on shortening.

Historically, midshaft clavicle fractures have been treated conservatively with low nonunion rate, but without including displacement factors [1, 19]. More recently, some studies have shown less satisfactory results with nonunion rates up to 15%, depending on the displacement, with an impact on the function of the shoulder [8, 20]. This has led to highlight several risk factors for poor results of conservative treatment [21]. Among those, shortening has been shown to have an impact on the union rate, function, and personal satisfaction [4, 22–24]. McKee et al. showed an inverse relationship between shortening and endurance in abduction for a threshold of 20 mm of shortening [23]. This trend was confirmed by Lazarides et al. which showed poor functional results with a threshold of 14 mm for women and 18 mm for men [22]. It seems that the clavicle shortening is reflected throughout the shoulder with impaired scapula-thoracic and glenoid alignment, which negatively affects mobility and muscle tension in the peri-scapular musculature [25–29].

Thus, the knowledge of the effect of shortening of clavicle fractures have led to more indication to osteosynthesis of these fractures. Better results were obtained for functional scores, accelerated return to work and overall satisfaction when a surgery was proposed for displaced fractures [22–24]. However, this led sometimes to the over-indication of surgery along with possible complications, such as infection and necessity of hardware removal.

To avoid over-indication, the goal of this study was to develop accurate measurements of clavicle fractures shortening in daily practice. CT is widely recognized in the literature as the gold-standard in shortening measurement [10, 16]. This study confirms the use of CT with virtual fragments reposition as the reference modality, with a non-significant minimal difference of 0.736 mm (95CI = − 2.51;3.98) compared to manual clavicle length measurements. However, in clinical practice, the surgeon is frequently limited to decision based on 2D imaging. Omid et al. assessed the measurement of standard radiographic shortening compared to CT [10]. Despite good reproducibility of radiological measurements, they were not significantly correlated with those on CT. Nevertheless, in this study, measurements were made on chest X-rays by measuring the length difference with the contralateral side. This methodology assuming symmetry between the two sides may be biased. Cadaveric and anthropological studies have shown side to side differences up to 15 to 20 mm [12, 13]. More recently, Cunningham et al. performed a radiological study comparing the length of the two clavicles on CT [30]. They demonstrated 28% asymmetry of more than 5 mm, of which 7% of more than 10 mm. This was confirmed by Hoogervorst et al., showing 30% of asymmetry over 5 mm when measured on CT [31].

In the present study, four measurement methods were evaluated based on single side imaging. The Silva et al. and the HUG methods were significantly correlated to 3D measuring with mean difference of − 1.02 mm and − 2.03 mm, respectively, therefore these may be used clinically. This is confirmed by the Bland-Altman analysis as shown in Fig. 3. These two methods are those whose differences with CT are closest to zero with minimal dispersion. Jones et al. measured shortening on a single incidence [32]. Their analysis focused solely on the reproducibility of the measurements (without specifying the technique). Their results showed a low inter-observer correlation. On the opposite, our study showed a very good reproducibility with ICC from 0.84 to 0.98. However, the methods of Jeray and Smekal et al. showed a limited correlation with the CT measurements, and we do not recommend their use for clinical decision.

The technique based on the study of Silva et al. showed measurements performed on anteroposterior incidences of fracture of the middle third of clavicle of pediatric patients [15]. The analysis focused solely on the reproducibility of measures, without correlation with CT. The inter-observer reliability was not good enough to allow its clinical use (ICC 0.69 to 0.74). In our study, we applied this method to adult clavicles with good reliability (ICC 0.97). When compared to 3D CT reconstructions, the results were also reliable. The HUG method was the measurement showing the best correlation with 3D-VR among the four radiographic methods evaluated.

In addition, the method of Silva et al. and the HUG method were the ones that minimized the most the diagnostic error. A difference exceeding 20 mm of shortening in comparison with 3D-VR was found in 5–10% (two observers) with the HUG method and 15% with the Silva et al. method. Use of 3D-VR technique could hence avoid unnecessary surgical treatment and related complications for the patients.

Strength of our study lies in the use of 3D CT reconstruction. Indeed, most of the studies comparing standard radiographic measurement with CT remained in the 2D plane [10, 16]. However, 3D reconstruction provides the advantage to standardize the reference points for the measurement of length, and thus to minimize the error rate. Even if the collimation used on a total body CT for a polytrauma patient is different from that of a clavicle, because of the wide field of view studied (soft tissue and bony structures), the infra-millimetric difference between cut of 1.25 mm and 0.3 mm probably does not affect the measurement significantly, especially on 3D-VR reconstructions. To our knowledge, this study is the first to evaluate shortening with 3D reconstructions and virtual repositioning of fragments, simulating surgical management. Moreover, all studies comparing 2D vs. 3D measurements were done on consolidated or non-fractured clavicle. Our study is the first to assess fractured clavicle.

Shortening has usually been measured by standard radiographs. Studies have shown that image distortion due to radiological image incidence modifies the amount of measured shortening, leading to changes from operative to nonoperative indication in 33.9% of the cases [33, 34]. With our study confirming the accuracy of the use of CT and 3D reconstruction with virtual reposition, this technique could be a valuable alternative, opening the question on the use of low-dose CT for clavicle fracture.

## Conclusion

Midshaft clavicle fracture shortening measurement according to HUG and Silva et al. methods were the most accurate 2D radiographic methods in our study. They allow for approximation of shortening with a moderate risk of overestimation and excessive surgical indication. Three-dimensional shortening with virtual fragments repositions (3D-VR) lead to measurements 5 mm within the confidence interval. We recommend its use if CT imaging is available. Development of Low-dose CT protocol could allow for implementation of this method without increasing patient’s exposure to irradiations.

## Data Availability

The datasets used and analyzed during the current study are available from the corresponding author on reasonable request.

## Abbreviations

3D-VR: Three-dimensional reconstruction and virtual reposition of bone fragments
CT: Computed tomography
ICC: Intra-Class Correlation coefficient

## References

[1] Neer CS (1960). Nonunion of the clavicle. JAMA.

[2] Robinson CM (1998). Fractures of the clavicle in the adult: epidemiology and classification. J Bone Joint Surg Br..

[3] Postacchini F, Gumina S, De Santis P, Albo F (2002). Epidemiology of clavicle fractures. J Shoulder Elb Surg.

[4] Canadian Orthopaedic Trauma Society (2007). Nonoperative treatment compared with plate fixation of displaced Midshaft clavicular fractures: a multicenter, randomized clinical trial. J Bone Joint Surg Am.

[5] Nowak J, Holgersson M, Larsson S (2004). Can we predict long-term sequelae after fractures of the clavicle based on initial findings? A prospective study with nine to ten years of follow-up. J Shoulder Elb Surg.

[6] van der Meijden OA, Gaskill TR, Millett PJ (2012). Treatment of clavicle fractures: current concepts review. J Shoulder Elb Surg.

[7] Jones SD, Bravman JT (2021). Midshaft clavicle fractures—when to operate. Ann Joint.

[8] Hill JM, McGuire MH, Crosby LA (1997). Closed treatment of displaced middle-third fractures of the clavicle gives poor results. J Bone Joint Surg Br.

[9] Smekal V, Irenberger A, Struve P, Wambacher M, Krappinger D, Kralinger FS (2009). Elastic stable intramedullary nailing versus nonoperative treatment of displaced Midshaft clavicular fractures-a randomized, controlled. Clinical Trial J Orthop Trauma.

[10] Omid R, Kidd C, Yi A, Villacis D, White E (2016). Measurement of clavicle fracture shortening using computed tomography and chest radiography. Clin Orthop Surg.

[11] Thorsmark AH, Muhareb Udby P, Ban I, Frich LH (2017). Bone shortening of clavicular fractures: comparison of measurement methods. BMC Musculoskelet Disord.

[12] Auerbach BM, Raxter MH (2008). Patterns of clavicular bilateral asymmetry in relation to the humerus: variation among humans. J Hum Evol.

[13] McCormick WF, Stewart JH, Greene H (1991). Sexing of human clavicles using length and circumference measurements. Am J Forensic Mad Pathol.

[14] Jeray KJ (2007). Acute midshaft clavicular fracture. J Am Acad Orthop Surg.

[15] Silva SR, Fox J, Speers M, Seeley M, Bovid K, Farley FA (2013). Reliability of measurements of clavicle shaft fracture shortening in adolescents. J Pediatr Orthop.

[16] Smekal V, Deml C, Irenberger A, Niederwanger C, Lutz M, Blauth M (2008). Length determination in midshaft clavicle fractures: validation of measurement. J Orthop Trauma.

[17] Krouwer JS (2008). Why bland–Altman plots should useX, not (Y+X)/2 whenX is a reference method. Statist Med.

[18] Shrout PE, Fleiss JL (1979). Intraclass correlations: uses in assessing rater reliability. Psychol Bull.

[19] Rowe CR (1968). An atlas of anatomy and treatment of midclavicular fractures. Clin Orthop Relat Res.

[20] Zlowodzki M, Zelle BA, Cole PA, Jeray K, McKee MD (2005). Treatment of acute midshaft clavicle fractures: systematic review of 2144 fractures: on behalf of the evidence-based orthopaedic trauma working group. J Orthop Trauma.

[21] Jørgensen A, Troelsen A, Ban I (2014). Predictors associated with nonunion and symptomatic malunion following non-operative treatment of displaced midshaft clavicle fractures—a systematic review of the literature. Int Orthop.

[22] Lazarides S, Zafiropoulos G (2006). Conservative treatment of fractures at the middle third of the clavicle: the relevance of shortening and clinical outcome. J Shoulder Elb Surg.

[23] McKee MD (2006). Deficits following nonoperative treatment of displaced midshaft clavicular fractures. J Bone Joint Surg Am.

[24] Wick M, Müller EJ, Kollig E, Muhr G (2001). Midshaft fractures of the clavicle with a shortening of more than 2 cm predispose to nonunion. Arch Orthop Trauma Surg.

[25] Andermahr J, Jubel A, Elsner A, Prokop A, Tsikaras P, Jupiter J (2006). Malunion of the clavicle causes significant glenoid malposition: a quantitative anatomic investigation. Surg Radiol Anat.

[26] Hillen RJ, Bolsterlee B, Veeger DHEJ (2016). The biomechanical effect of clavicular shortening on shoulder muscle function, a simulation study. Clin Biomech.

[27] Ledger M, Leeks N, Ackland T, Wang A (2005). Short malunions of the clavicle: an anatomic and functional study. J Shoulder Elb Surg.

[28] Matsumura N, Ikegami H, Nakamichi N, Nakamura T, Nagura T, Imanishi N (2010). Effect of shortening deformity of the clavicle on scapular kinematics: a cadaveric study. Am J Sports Med.

[29] Matsumura N, Nakamichi N, Ikegami H, Nagura T, Imanishi N, Aiso S (2013). The function of the clavicle on scapular motion: a cadaveric study. J Shoulder Elb Surg.

[30] Cunningham BP, McLaren A, Richardson M, McLemore R. Clavicular length: the assumption of symmetry. Orthopedics. 2013; cité 4 août 2021;36. Disponible sur: http://journals.healio.com/doi/10.3928/01477447-20130222-24. 10.3928/01477447-20130222-24 23464955

[31] Hoogervorst P, Appalsamy A, Franken S, van Kampen A, Hannink G (2018). Quantifying shortening of the fractured clavicle assuming clavicular symmetry is unreliable. Arch Orthop Trauma Surg.

[32] Jones GL, Bishop JY, Lewis B, Pedroza AD, Baumgarten K, MOON shoulder group (2014). Intraobserver and Interobserver agreement in the classification and treatment of Midshaft clavicle fractures. Am J Sports Med.

[33] Hoogervorst P, Appalsamy A, Meijer D, Doornberg JN, van Kampen A, Hannink G (2019). Does altering projection of the fractured clavicle change treatment strategy?. J Shoulder Elb Surg.

[34] Hoogervorst P, Appalsamy A, van Geene AR, Franken S, van Kampen A, Hannink G (2018). Influence of x-ray direction on measuring shortening of the fractured clavicle. J Shoulder Elb Surg.

